# Perceived usefulness and ease of use of fundoscopy by medical students: a randomised crossover trial of six technologies (eFOCUS 1)

**DOI:** 10.1186/s12909-020-02469-8

**Published:** 2021-01-08

**Authors:** H. P. Dunn, C. J. Kang, S. Marks, J. L. Witherow, S. M. Dunn, P. R. Healey, A. J. White

**Affiliations:** 1 Faculty of Medicine & Health, University of Sydney, Sydney, Australia; 2 Department of Ophthalmology, Westmead Hospital, Sydney, Australia; 3 Discipline of Physiotherapy, Faculty of Medicine & Health Services, Macquarie University, Sydney, Australia; 4 Centre for Vision Research, Westmead Institute for Medical Research, University of Sydney, Sydney, Australia

**Keywords:** Fundoscopy, Smartphone, Education, Non mydriatic camera, Direct ophthalmoscope

## Abstract

**Background:**

Fundoscopy outside ophthalmology is in decline, and the technical demands of the traditional direct ophthalmoscope examination are likely contributing. Alternative fundoscopy technologies are increasingly available, yet valid comparisons between fundoscopy technologies are lacking. We aimed to assess medical students’ perceptions of usefulness and ease of use of traditional and contemporary fundus-viewing technologies including smartphone fundoscopy.

**Methods:**

One hundred forty-six second-year medical students participated in a cross-sectional, randomised, cross-over study of fundoscopy methods. Medical students completed small group training sessions using six current fundoscopy technologies including: a non-mydriatic fundus camera; two types of direct fundoscopy; and three types of smartphone fundoscopy. A novel survey of perceived usefulness and ease of use was then completed by students.

**Results:**

Repeated-measures ANOVA found students rated both the perceived usefulness (*p*< 0.001) and ease of use (*p*< 0.001) of smartphone fundoscopy significantly higher than both the non-mydriatic camera and direct fundoscopy.

**Conclusions:**

Smartphone fundoscopy was found to be significantly more useful and easier to use than other modalities. Educators should optimise student access to novel fundoscopy technologies such as smartphone fundoscopy which may mitigate the technical challenges of fundoscopy and reinvigorate use of this valuable clinical examination.

**Supplementary Information:**

The online version contains supplementary material available at 10.1186/s12909-020-02469-8.

## Background

Fundoscopy remains one of the great teaching challenges despite being a core skill for clinicians [1, 2], recognised by the International Council of Ophthalmology as one of seven basic ophthalmic medical education competencies [3]. Fundoscopy offers a non-invasive view of the central nervous system, revealing clinical signs of raised intracranial pressure [4, 5], end-organ damage from hypertension and diabetes [6, 7] and even the risk of stroke [8, 9]. Despite the clinical benefits of fundoscopy for patients, it is infrequently performed in routine clinical practice across community internists [10], hospital physicians [11], emergency physicians [12] and even neurologists [13]. Current fundoscopy practice is not only infrequent but poorly reliable. Bruce et al. found that 13% of ED patients who warranted fundoscopy had acute life or vision-threatening fundus pathology, yet none of these findings were identified by emergency physicians using the traditional direct ophthalmoscope (TDO) [12].

While current clinical practice is limited by the shortcomings of TDO, novel fundus imaging technologies offer a potential solution. The changed optics of the panoptic ophthalmoscope offer a wider field of view than the TDO, probably contributing to higher diagnostic accuracy in some studies [14–16]. Fundus photography is closer to the gold standard of dilated expert examination than TDO [17, 18]. Where novel technologies create a digital fundus image, the difficulties of interpreting findings may be mitigated [19] by the ability to modify, review and transmit the fundus image. Technological improvements to fundus imaging utilising the ubiquitous smartphone could improve device availability [20–22] alongside reduced technical barriers, and have shown similar diagnostic accuracy to fundus photography in some studies [23]. In the era of COVID-19 social distancing, the physical proximity of patient and clinician when using the TDO is potentially hazardous to both [24]. Despite the burgeoning availability of fundus-viewing technologies [25] and fundoscopy simulators, there is a paucity of comparative effectiveness studies [26] and a lack of validated tools for comparing their performance.

The present decline in fundoscopy has been attributed to technical difficulties in performing traditional ophthalmoscopy, compounded by the challenge of interpreting clinical findings [27]. From the perspective of doctors choosing to examine the fundus or not, these causes align respectively with perceived ease of use (PEOU) and perceived usefulness (PU), as described in the Technology Acceptance Model [28]. Perceived ease of use and usefulness have been repeatedly shown to predict future use of technologies [28–31], and underlie attitudes and behaviours towards health technology [32–35]. If fundoscopy is easier to perform and interpret, uptake by medical students and clinicians will likely improve. This should lead to better patient outcomes, so long as future studies concurrently demonstrate acceptable diagnostic accuracy of novel fundoscopy devices.

In this study we aimed to develop a survey of perceived ease of use and usefulness relevant to fundoscopy; and to measure these factors for commercially available fundoscopy technologies. We hypothesised that perceived ease of use and usefulness would be significantly different between technologies, and that smartphone fundoscopy would outperform TDO.

## Methods

This study adhered to the Declaration of Helsinki and Ethics Committee approval was obtained. Written informed consent was obtained from participants.

### Study design

Cross-sectional, randomised, cross-over study.

### Setting & participants

343 second-year medical students from the University of Sydney undertaking a mandatory ophthalmology training day at the Sydney Eye Hospital were offered study participation. No prior practical or theoretical ophthalmic teaching was conducted in their degree.

### Educational intervention and fundoscopy devices

Training started with a 20-min eye anatomy and examination talk with a video overview of TDO technique. Students were asked to have one pupil dilated with tropicamide 0.5% to facilitate fundus examination. All 343 students were randomised into groups of 12–16 students using a computer-generated random number given out on arrival. Each group commenced with a different 10-min station to avoid any learning bias from the order of instruction, and rotated through six stations: (1) NMC with combined ocular coherence tomography [Topcon, 3D OCT-1 Maestro, Tokyo, Japan]; (2) coaxial traditional direct ophthalmoscope (TDO) [Welch Allyn, Macquarie Park, NSW, Australia]; (3) Panoptic Ophthalmoscope [Welch Allyn, Macquarie Park, NSW, Australia], and; smartphone fundoscopy with (4) Panoptic + iExaminer [Welch Allyn, Macquarie Park, NSW, Australia]; (5) D-eye [Padova, Italy]; and (6) a prototype smartphone adaptor each attached to an iPhone 5 or 6 [Apple Inc., Cupertino, CA, USA] (see Additional file 1 for device characteristics). An ophthalmologist and a technical assistant were present at each station providing standardised device instructions and assistance if required. Students then examined each other with each device.

### Survey development

Davis et al’s 12-item PU & PEOU survey [28] was modified for application to medical devices, with a target population of medical students and doctors. The survey was used for content validity and the original six domains were adapted for both PEOU and PU [36]. Candidate questions were then optimised via independent verification by the authors and three non-participant medical students, to generate the final wording (Table 1). The authors had diverse backgrounds in medicine (HD, CK, SD, AW, PH), ophthalmology (HD, AW, PH), psychology and qualitative research (HD, SD), and biomedical and computer engineering (SM). A 5-point Likert scale was used from 1 = ‘Unlikely’ to 5 = ‘Likely’, yielding a score range of 6 to 30 for usefulness and PEOU.

**Table 1:** 6-item survey of usefulness and ease of use showing the original constructs and the final modified wording

Scale items	Original construct	Modified wording
**Usefulness**		
**1**	Work more quickly	Using the device in my clinical placements would enable me to accomplish tasks more quickly
**2**	Job performance	Using the device would improve my clinical performance
**3**	Increase productivity	Using the device in my clinical placement would increase my productivity
**4**	Effectiveness	Using the device would enhance my effectiveness
**5**	Makes job easier	Using the device would make it easier to do my clinical placements
**6**	Useful	I would find the device useful in my clinical placements
**Ease of Use**		
**1**	Easy to learn	Learning to operate the device would be easy for me
**2**	Controllable	I would find it easy to get the device to do what I want it to do
**3**	Clear & understandable	My interaction with the device would be straightforward
**4**	Flexible	I would find the device to be flexible to use in different clinical scenarios
**5**	Easy to become skillful	It would be easy for me to become skillful at using the device
**6**	Easy to use	I would find the device easy to use

In order to maximise response rates, questionnaire length was streamlined [37] and fundoscopy technologies were grouped as shown below:

1. Non-Mydriatic Camera (NMC);
2. Direct fundoscopy comprising TDO and Panoptic, and;
3. Smartphone fundoscopy comprising D-Eye and iExaminer.

The prototype was excluded as it was in early development and not commercially available.

### Survey

After each station students rated: the quality of training; their perceived ease of viewing the fundus; and confidence with the equipment. At the end of the training day, students were also asked to score the modalities of fundoscopy according to PU and PEOU; and how frequently they would examine the fundus during future general examinations (see Additional file 2).

### Outcomes

The primary outcomes were the perceived usefulness and ease of use scores for the technology groups. Secondary outcomes included confidence to view the fundus, and self-reported future fundoscopy practice patterns.

### Analysis

Statistical analysis was performed using SPSS (version 15.0; SPSS Inc., Chicago, IL). Continuous data were reported as mean and 95% confidence interval (CI), and categorical data as percentages. Statistical significance was set at α = 0.05. Survey scores for different fundoscopy technologies were examined using one-way repeated measures analysis of variance (ANOVA) and post-hoc comparisons performed with Bonferroni correction. Analysis of each survey question included results from participants with complete data, who recorded scores for all relevant fundoscopy technologies. Internal consistency of the items assessing PU and PEOU surveys was calculated using Cronbach’s alpha. Internal consistency of a psychometric score is generally rated well if the Cronbach alpha is between 0.7 and 0.95 [38].

## Results

### Baseline characteristics

Four training sessions were held over 2 days in May 2017 with a total of 343 students, of whom 146 completed the survey (43% response rate). The median age was 24 (IQR 22–26) and 78 (53%) were women. This was similar to the entire cohort amongst whom 47% were female (χ^2^=1.473, df=1, *p*=0.22). Twelve participants (8%) had some previous ophthalmology or optometry training. All 146 participants (100%) owned a smartphone.

**Fig. 1:**
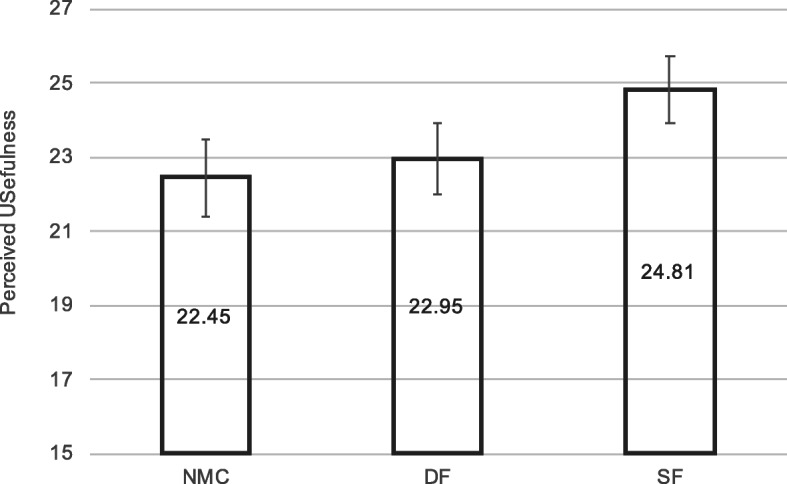
Mean scores of perceived usefulness by students for NMC, DF (comprising the traditional direct ophthalmoscope and panoptic ophthalmoscope), and SF (comprising iExaminer and D-eye). Error bars depict 95% confidence interval

Perceived usefulness scores were significantly different between different fundoscopy modalities (repeated-measures ANOVA *N*=100, F (2,198)=8.90, *p*< 0.001). Post-hoc pairwise comparisons showed SF was significantly more useful compared to NMC or direct fundoscopy (*p*< 0.001 and *p*=0.006 respectively) (Fig. 1). There was no significant difference between NMC and direct fundoscopy.

**Fig. 2:**
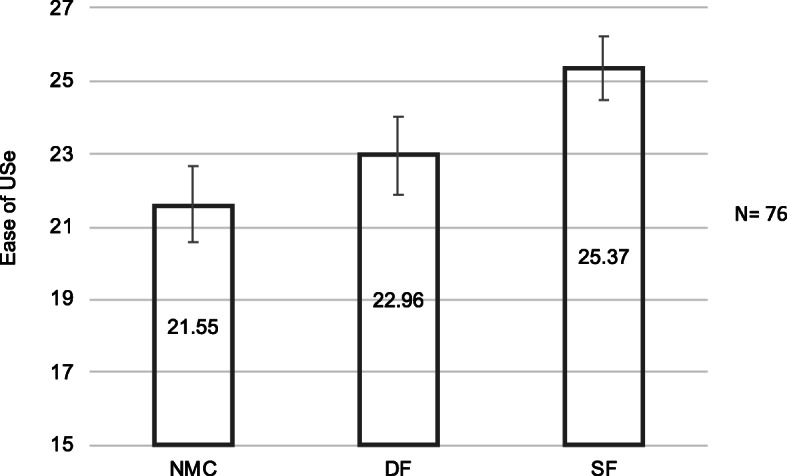
Mean scores of perceived ease of use by students for non-mydriatic camera (NMC), direct fundoscopy (DF, comprising the traditional direct ophthalmoscope and panoptic ophthalmoscope), and smartphone fundoscopy (SF, comprising iExaminer and D-eye). Error bars show 95% confidence interval

Similarly, PEOU scores were significantly different between different fundoscopy modalities (repeated-measures ANOVA with Huynh-Feldt correction, *N*=93, F (2,184)=16.865, *p*< 0.001). Post-hoc pairwise comparisons showed SF was rated significantly easier to use than NMC (*p*< 0.001) or direct fundoscopy (*p*< 0.001) (Fig. 2). There was no significant difference between NMC and direct fundoscopy (*p*=0.207).

The modified PUF and PEOU scores were found to have strong internal consistency. Analysis for the PUF scores for NMC, direct fundoscopy and SF found a Crohnbach’s alpha of 0.91, 0.93 and 0.91 respectively. The Crohnbach’s alpha for the PEOU scores for NMC, direct fundoscopy and SF were 0.94, 0.95 and 0.94 respectively.

There were no observed interaction effects of participants’ age, gender, previous ophthalmic training on PEOU or PUF scores (repeated-measures ANOVA, all *p*> 0.05).

**Fig. 3:**
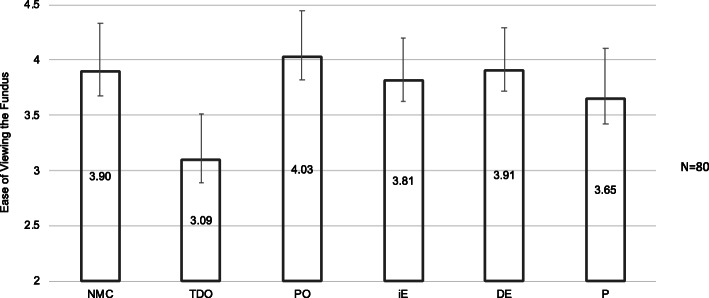
Mean ease of viewing the fundus by students. (NMC = non-mydriatic camera; TDO = traditional direct ophthalmoscope; PO = Panoptic ophthalmoscope; iE = iExaminer; DE = D-eye; P = prototype). Error bars depict 95% confidence interval

Scores for ease of viewing the fundus were significantly different between modalities (repeated measures ANOVA with Greenhouse- Geisser correction, *N*=76, F (3.6375)= 12.70, *p*< 0.001). Post-hoc pairwise comparisons showed students found viewing the fundus significantly harder with the TDO than all modalities (all *p*< 0.007) except the prototype (*p*=0.092). However, there were no significant differences between any other modalities (see Fig. 3).

**Fig. 4:**
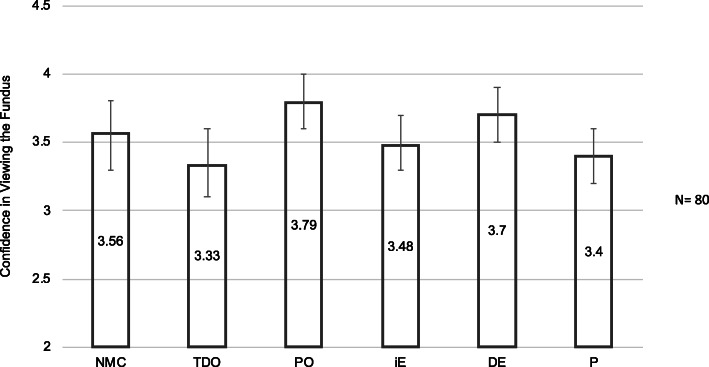
Mean confidence to view the fundus by students. (NMC = non-mydriatic camera; TDO = traditional direct ophthalmoscope; PO = Panoptic ophthalmoscope; iE = iExaminer; DE = D-eye; P = prototype). Error bars depict 95% confidence interval

Scores for confidence to view the fundus were also significantly different between modalities (repeated measures ANOVA with Greenhouse- Geisser correction, *N*=80, F (3.7292)= 4.70, *p*=0.002). Post-hoc testing found students were significantly less confident using the TDO than the Panoptic (*p*=0.001) and less confident with the TDO than the D-eye but this result did not reach significance (mean difference − 0.375, *p*=0.054). Students were more confident using the Panoptic than the prototype (*p*=0.019). There were no significant differences between NMC, Panoptic and the other smartphone modalities (DE and iE) (Fig. 4).

There was no difference in the perceived quality of training between modalities (one-way ANOVA *N*=85, F (2, 15)=1.50, *p*=0.19). Eighty-five participants (58%) stated that they would perform fundoscopy as a regular part of their general examination if not asked to do so by their supervisor.

## Discussion

This is the first study to develop validated measures of perceived ease of use and usefulness for fundoscopy. The results support our hypothesis that medical students perceive smartphone fundoscopy to be significantly more useful and easier to use than direct ophthalmoscopy. Furthermore, students found viewing the fundus significantly more difficult using the traditional direct ophthalmoscope than all other commercial technologies.

### Perceived ease of use

Medical student curricula are currently so crowded that little time is offered to ophthalmic training [39], and less still to the complex skill of fundoscopy. Fundoscopy technologies should therefore be as easy to operate as possible. We demonstrated that smartphone fundoscopy was significantly easier to use than direct fundoscopy or NMC (*p*< 0.001). Furthermore, students found viewing the fundus significantly harder with a TDO than any other commercial modality (*p*=0.002). Whilst other studies which have shown NMC to be easy to use by non-expert operators [13, 40], we found no significant differences between the NMC, D-Eye, iExaminer or Panoptic. These latter four devices share some technical advantages over TDO including a wider field of view, less proximity to the patient and the potential to store and review digital images.

The difficulty of using a TDO in our study is consistent with previous literature [18, 41, 42]. A study of 101 medical students conducted after our trial found TDO was harder to use than the smartphone-mounted D-Eye on an unvalidated Likert scale [43]. Mamtora et al. found smartphone fundoscopy improved the accuracy and quality of fundal examinations by medical students when compared with TDO [44]. Mandal and colleagues developed an ease of use score for fundoscopy based on the capacity to identify anatomical details [45], but the methodology for developing this scale was not reported, and no psychometric validation was performed. To our knowledge, our study is the first to report a validated score of ease of use or usefulness for fundoscopy.

### Perceived usefulness

We found that students rated the usefulness of smartphone fundoscopy significantly greater than both NMC and direct fundoscopy (mean scores 24.81, 22.45, 22.95 respectively; *p*< 0.001). Perceived usefulness consistently modifies new technology usage over time, whereas perceived ease of use appears to have more influence on early uptake [29, 30]. Correspondingly, the perceived impact of fundoscopy on patient management may drive usage more than ease of use. Hence usefulness is arguably the central factor in changing clinicians’ attitudes and behaviour. Indeed, some experts have suggested that medical school curricula should focus on training students to interpret fundus photographs rather than the vagaries of the TDO [2, 46]. A study of first-year medical students found 70% preferred fundus photographs over TDO in clinical practice and were more accurate at diagnosing pathology using photographs [47], with differences sustained after 1 year [41].

Doubtless there are factors beyond perceived usefulness and ease of use which account for fundoscopy practice patterns. However, these two factors consistently account for approximately 40% of the variance in both intended usage and actual behavior [30], which is greater than any other models of technology use [48]. Moreover, previous educational interventions to improve fundoscopy use which ignored these factors have been largely unsuccessful. Access alone was insufficient, as ownership of a portable direct fundoscopy device did not improve frequency or accuracy of fundoscopy in a randomised controlled trial of 42 students [42]. Retraining students in fundoscopy skills improved diagnostic accuracy but failed to improve the rate of documented fundoscopy in a three-year prospective educational study [49].

The emergence of portable NMCs, telecommunications and smartphone technology have driven an exponential increase in the availability of digital fundus imaging devices [25]. As smartphone fundoscopy image quality improves and NMCs become cheaper and more portable, the clinical usefulness of fundoscopy is likely to increase. A digital fundus image unlocks the capability of telehealth to send the image for specialist advice [50] and increasingly to process the image through artificial intelligence for decision support [51], thus optimising patients’ health and the cost-benefit of the examination. For students training in fundoscopy, a digital solution also removes the pressure of time when interpreting findings, limits patient discomfort when reviewing findings, and allows instructors to view and provide feedback on student performance, which is proven to improve results [52]. Many digital fundoscopy modalities, including all commercial devices in this study, have image storage solutions compliant with patient data security requirements. Once the fundus can be reliably visualised, the success of a telehealth intervention still relies on the motivation of physicians to change their clinical practices [53]. Hence, further fundoscopy education is needed to improve the clinical interpretation and implementation of fundus findings.

### Confidence

Limited confidence with fundoscopy examination has been widely reported, and identified as a significant barrier to usage [42, 47, 54]. We found after brief training that medical students were significantly less confident viewing the fundus using the traditional ophthalmoscope (*p*=0.001) or prototype (*p*=0.019) than the Panoptic, despite additional instruction for TDO. We found no significant differences in confidence between the novel fundoscopy technologies NMC, D-Eye, iExaminer or Panoptic.

Confidence alone does not predict clinical utility, and perceived usefulness and ease of use are likely better predictors of future fundoscopy practice. Indeed, a study assessing TDO proficiency found students who were incorrect when matching a patient’s fundus to a grid of fundus photographs were more confident in their decision than those who were correct [55].

### Strengths

Our study was strengthened by the use of: a proven theoretical framework using the Technology Acceptance Model [30]; a survey validated to predict future use of novel technologies [28] and validated in health contexts [32–35], and; rigorous survey development methodology [38]. This is reflected in the excellent internal consistency [38] of the measures of PU and PEOU (Crohnbach alphas 0.91–0.95).

Student groups were randomised to commence with different fundoscopy technologies, thus avoiding any effect of the order of instruction. Our findings appear attributable to factors inherent in the technologies, rather than the instruction methods, as there was no significant difference in perceived training quality, and no interaction effects of participants’ age, gender, or previous ophthalmic training on PEOU or PU scores.

### Limitations

Our study has some notable limitations. The response rate was 43% (146/343), although the known characteristics of our participants were not significantly different to the entire student cohort. Our participants were second-year medical students with limited clinical experience whose perceptions of usefulness are likely to differ from experienced clinicians. However, perceived ease of use is less likely to be confounded by clinical experience and may therefore have a more general application. This could be tested by future studies exploring perceived ease of use and usefulness amongst experienced clinicians.

Although we found relatively small absolute differences in perceived ease of use and usefulness scores between modalities, the survey we employed has proven strong discriminant validity for distinguishing similar technologies [56].

Aspects of our protocol could have biased students’ responses. Students were shown an instructional video for the use of TDO only, which may have increased their capability and comfort with the TDO examination, or anchored their perceptions of appropriate technology for patient examination [57]. Our PEOU and PU scores collated opinions regarding fundoscopy technologies into groups of smartphone fundoscopy, direct fundoscopy and the non-mydriatic camera, in order to assess general distinctions between these groups, and to streamline the length of the survey aiming to improve response rate [37]. However this raises a possible unknown effect of grouping. For example, the TDO and Panoptic were collated under the umbrella term ‘direct fundoscopy’, yet when surveyed individually the Panoptic outperformed the TDO in all secondary outcomes. Future studies may minimise group confounding by including fewer comparative technologies and representing each individually within the survey.

Whilst our study showed that medical students perceived smartphone fundoscopy as more useful and easier to use than the TDO or a non-mydriatic camera, we did not compare their diagnostic accuracy when using these tools. Further comparative studies will be required to avoid the possibility that an easy to use device may be marred by diagnostic inaccuracy.

## Conclusion

The current clinical practice of fundoscopy is inappropriately infrequent [10–13]. If fundoscopy is easier to perform and interpret, students may find it easier to train and clinicians more likely to perform fundoscopy in clinical practice. Perceived usefulness and ease of use are the best predictors of future technology use [29–31]. Hence we developed the first validated survey of these factors for fundoscopy and compared medical students’ perceptions of current fundoscopy technologies. Our findings demonstrate that novel fundoscopy technologies including smartphone fundoscopy are perceived as more useful and easier to use than traditional direct ophthalmoscopy. We suggest that physicians and students should have better access to novel fundoscopy technologies. Further studies will need to compare fundoscopic technologies with regards to diagnostic accuracy, educational outcomes for students, fundoscopy utilisation by clinicians, and ultimately clinical outcomes for patients.

## Supplementary Information

**Additional file 1.** Characteristics of fundoscopy technologies used in this study. a = details supplied by distributors; b = also requires smartphone; c = also requires PO; NMC = non-mydriatic camera; TDO = traditional direct ophthalmoscope; PO = Panoptic ophthalmoscope; iE = iExaminer; DE = D-eye; P = prototype).

**Additional file 2.** Student training questionnaire.

## Data Availability

The datasets generated and analysed during the current study are available from the corresponding author on reasonable request.

## Abbreviations

DE: D-eye
DF: Direct fundoscopy
iE: iExaminer
NMC: Non-mydriatic camera
P: Prototype
PEOU: Perceived ease of use
PO: Panoptic ophthalmoscope
PU: Perceived usefulness
SF: Smartphone fundoscopy
TDO: Traditional direct ophthalmoscopy
