# Supplementary material for: Perceived usefulness and ease of use of fundoscopy by medical students: a randomised crossover trial of six technologies (eFOCUS 1)
Source: BMC Med Educ. 2021 Jan 8;21:41. doi: 10.1186/s12909-020-02469-8 (PMC7793394; doi:10.1186/s12909-020-02469-8)
Supplement: Supplementary file 1 — Additional file 1. Characteristics of fundoscopy technologies used in this study. a = details supplied by distributors; b = also requires smartphone; c = also requires PO; NMC = non-mydriatic camera; TDO = traditional direct ophthalmoscope; PO = Panoptic ophthalmoscope; iE = iExaminer; DE = D-eye; P = prototype). [file 12909_2020_2469_MOESM1_ESM.docx]

|  | **Manufacturer** | **Field of view** | **Digital image capture** | **Cost ($AUD)** | **Dimensions (W x D x H cm)** | **Weight (kg)** |
| --- | --- | --- | --- | --- | --- | --- |
| **NMC** | Topcon ^a^ | 45°/30° | Yes | 74 960 | 30.7–44.3× 47.2- 66.8 × 51.8–72.2 | 21 |
| **TDO** | Welch Allyn ^a^ | 5° | No | 865.05 | 5.1 × 8.6 × 4.5 | 0.35 |
| **PO** | Welch Allyn ^a^ | 25° | Possible (can have an iExaminer adaptor fitted) | 1443.70 | 19.8 × 11.8 × 5.4 | 0.3 |
| **iE** | Welch Allyn ^a,b,c^ | 25° | Yes | 69.4 | 29 x 12.1 x 6 | 0.3 |
| **DE** | D-Eye ^a,b^ | 10° | Yes | 631 | 5 × 2.1 × 0.97 | 0.027 |
| **P** | Sydney Scientific ^a,b^ | 5-8° | Yes | 43 | 2.5 x 2.5 | 0.019 |

a = details supplied by distributors; b = also requires smartphone; c = also requires PO; NMC = non- mydriatic camera; TDO = traditional direct ophthalmoscope; PO = Panoptic ophthalmoscope; iE = iExaminer; DE = D- eye; P = prototype).
