# Supplementary material for: Perceived usefulness and ease of use of fundoscopy by medical students: a randomised crossover trial of six technologies (eFOCUS 1)
Source: BMC Med Educ. 2021 Jan 8;21:41. doi: 10.1186/s12909-020-02469-8 (PMC7793394; doi:10.1186/s12909-020-02469-8)
Supplement: Supplementary file 2 — Additional file 2. Student training questionnaire. [file 12909_2020_2469_MOESM2_ESM.docx]

PRE-TRAINING

| 1. What is your Student ID? |  |
| --- | --- |
| 1. Please provide the following demographic information: | Male Female (*Circle one)* |
|  | Age (in years) ………………………… |
| 1. Have you had any previous ophthalmology/optometry training? | No Yes *(Please describe)*  ………………………………………………………. |
| 1. Do you own a smartphone? | No Yes *(Which make/model?)*  ……………………………………………………… |
| 1. Did you complete the online training in funduscopy interpretation? | No Yes |
| 1. If you wish to be involved please provide your email address. | Email  ……………………………………………………… |

POST-TRAINING - **Immediately following each station** please answer the following questions honestly based on the training you have just completed.

1. Rate the quality of training you received at each station:

|  | Poor |  |  |  | Excellent |
| --- | --- | --- | --- | --- | --- |
| Non-mydriatic camera | 1 | 2 | 3 | 4 | 5 |
| Direct ophthalmoscope | 1 | 2 | 3 | 4 | 5 |
| Pan-ophthalmoscope | 1 | 2 | 3 | 4 | 5 |
| iExaminer | 1 | 2 | 3 | 4 | 5 |
| D-eye | 1 | 2 | 3 | 4 | 5 |
| **Prototype** | 1 | 2 | 3 | 4 | 5 |

1. On a scale from 1 to 5, rate the ease of viewing the ocular fundus using each technique. Please circle only ONE number.

|  | Very Difficult |  |  |  | Very easy |
| --- | --- | --- | --- | --- | --- |
| Non-mydriatic camera | 1 | 2 | 3 | 4 | 5 |
| Direct ophthalmoscope | 1 | 2 | 3 | 4 | 5 |
| Pan-ophthalmoscope | 1 | 2 | 3 | 4 | 5 |
| iExaminer | 1 | 2 | 3 | 4 | 5 |
| D-eye | 1 | 2 | 3 | 4 | 5 |
| **Prototype** | 1 | 2 | 3 | 4 | 5 |

1. On a scale from 1 to 5, rate your level of confidence in being able to view the fundus with each technique after today’s training session. Please circle only ONE number.

|  | No confidence |  |  |  | Very confident |
| --- | --- | --- | --- | --- | --- |
| Non-mydriatic camera | 1 | 2 | 3 | 4 | 5 |
| Direct ophthalmoscope | 1 | 2 | 3 | 4 | 5 |
| Pan-ophthalmoscope | 1 | 2 | 3 | 4 | 5 |
| iExaminer | 1 | 2 | 3 | 4 | 5 |
| D-eye | 1 | 2 | 3 | 4 | 5 |
| **Prototype** | 1 | 2 | 3 | 4 | 5 |

**Please complete the following questions AFTER COMPLETING ALL SESSIONS:**

1. If not specifically requested by your supervisor, would you perform funduscopy on a patient as part of a general physical examination?

Yes / No

1. Over the next year, how often do you think you will attempt to examine the ocular fundus on patients as part of your general exam? Please circle only ONE number.
   1. never
   2. 1-25% of the time
   3. 26-50% of the time
   4. 51-75% of the time
   5. 76-99% of the time
   6. always
2. Please indicate how likely you feel each statement would be true for each group of devices.

|  | Non-mydriatic camera | | | | | **Direct Ophthalmoscopy**  **(including Panoptic)** | | | | | **Smartphone Funduscopy**  **(D-eye & iExaminer)** | | | | |
| --- | --- | --- | --- | --- | --- | --- | --- | --- | --- | --- | --- | --- | --- | --- | --- |
|  | Unlikely | |  | Likely | | Unlikely | |  | Likely | | Unlikely | |  | Likely | |
| 1. Learning to operate the device would be easy for me | 1 | 2 | 3 | 4 | 5 | 1 | 2 | 3 | 4 | 5 | 1 | 2 | 3 | 4 | 5 |
| **2. I would find it easy to get the device to do what I want it to do** | 1 | 2 | 3 | 4 | 5 | 1 | 2 | 3 | 4 | 5 | 1 | 2 | 3 | 4 | 5 |
| 3. My interaction with the device would be straightforward | 1 | 2 | 3 | 4 | 5 | 1 | 2 | 3 | 4 | 5 | 1 | 2 | 3 | 4 | 5 |
| 4. I would find the device to be flexible to use in different clinical scenarios | 1 | 2 | 3 | 4 | 5 | 1 | 2 | 3 | 4 | 5 | 1 | 2 | 3 | 4 | 5 |
| 5. It would be easy for me to become skillful at using the device | 1 | 2 | 3 | 4 | 5 | 1 | 2 | 3 | 4 | 5 | 1 | 2 | 3 | 4 | 5 |
| 6. I would find the device easy to use | 1 | 2 | 3 | 4 | 5 | 1 | 2 | 3 | 4 | 5 | 1 | 2 | 3 | 4 | 5 |

|  | Non-mydriatic camera | | | | | **Direct Ophthalmoscopy**  **(including Panoptic)** | | | | | **Smartphone Funduscopy**  **(D-eye & iExaminer)** | | | | |
| --- | --- | --- | --- | --- | --- | --- | --- | --- | --- | --- | --- | --- | --- | --- | --- |
|  | Unlikely | |  | Likely | | Unlikely | |  | Likely | | Unlikely | |  | Likely | |
| 7. Using the device in my clinical placements would enable me to accomplish tasks more quickly | 1 | 2 | 3 | 4 | 5 | 1 | 2 | 3 | 4 | 5 | 1 | 2 | 3 | 4 | 5 |
| **8. Using the device would improve my clinical performance** | 1 | 2 | 3 | 4 | 5 | 1 | 2 | 3 | 4 | 5 | 1 | 2 | 3 | 4 | 5 |
| 9. Using the device in my clinical placement would increase my productivity | 1 | 2 | 3 | 4 | 5 | 1 | 2 | 3 | 4 | 5 | 1 | 2 | 3 | 4 | 5 |
| 10. Using the device would enhance my effectiveness | 1 | 2 | 3 | 4 | 5 | 1 | 2 | 3 | 4 | 5 | 1 | 2 | 3 | 4 | 5 |
| 11. Using the device would make it easier to do my clinical placements | 1 | 2 | 3 | 4 | 5 | 1 | 2 | 3 | 4 | 5 | 1 | 2 | 3 | 4 | 5 |
| 12. I would find the device useful in my clinical placements | 1 | 2 | 3 | 4 | 5 | 1 | 2 | 3 | 4 | 5 | 1 | 2 | 3 | 4 | 5 |
